# P-102. Six-Month Safety, Durability, and Cross-Neutralization of the SARS-CoV-2 XBB.1.5-Containing Vaccine

**DOI:** 10.1093/ofid/ofae631.309

**Published:** 2025-01-29

**Authors:** Frances Priddy, Nichole McGhee, Spyros Chalkias, Jordan L Whatley, Brandon Essink, Adam Brosz, Bethany Girard, Darby Wang, Elizabeth de Windt, Darin Edwards, Jing Feng, Angeline Sanders, David C Montefiori, Jacqueline Miller, Rituparna Das

**Affiliations:** Moderna, Inc., Cambridge, Massachusetts; Moderna, Inc., Cambridge, Massachusetts; Moderna, Inc., Cambridge, Massachusetts; Meridian Clinical Research, Baton Rouge, Louisiana; Meridian Clinical Research, Baton Rouge, Louisiana; Meridian Clinical Research, Baton Rouge, Louisiana; Moderna, Inc, Cambridge, Massachusetts; Moderna, Inc, Cambridge, Massachusetts; Moderna, Inc., Cambridge, Massachusetts; Moderna, Inc., Cambridge, Massachusetts; Moderna, Inc., Cambridge, Massachusetts; PPD, part of Thermo Fisher Scientific, Wilmington, North Carolina; Duke University Medical Center, Durham, North Carolina; Moderna, Inc., Cambridge, Massachusetts; Moderna, Inc., Cambridge, Massachusetts

## Abstract

**Background:**

Monovalent omicron XBB.1.5-containing vaccines were approved for use in 2023-2024 COVID-19 immunizations to better match emergent XBB variants. In an ongoing phase 2/3 study, the monovalent omicron XBB.1.5 vaccine (mRNA-1273.815) was previously shown to elicit robust and diverse neutralizing antibody (nAb) responses through 28 days after vaccination. Here, we present on 6-month safety and immunogenicity of mRNA-1273.815, including cross-neutralization of emergent variants.

**Figure:**
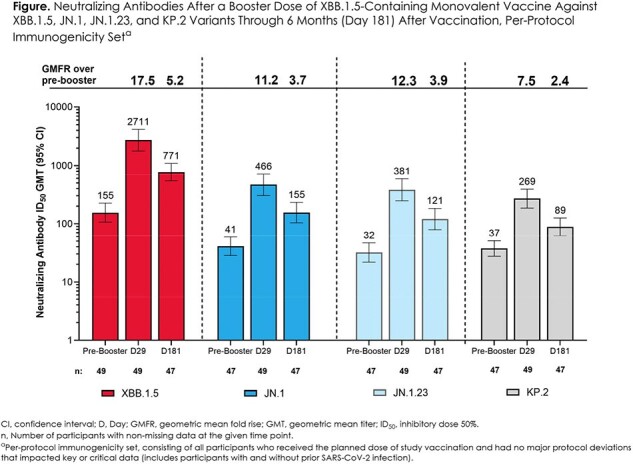

**Methods:**

This open-label, phase 2/3 study (NCT04927065) randomized participants (1:1) to receive a 50 µg dose of monovalent mRNA-1273.815 (50 µg omicron XBB.1.5 spike mRNA) or bivalent mRNA-1273.231 (25 µg omicron XBB.1.5 and 25 µg omicron BA.4/BA.5 spike mRNAs). Participants had received 4 prior doses of a COVID-19 vaccine (2-dose primary series and first booster of an original mRNA vaccine plus a booster of any bivalent mRNA omicron BA.4/BA.5-containing vaccine). Reactogenicity and safety through Day 181 (6 months) was a primary objective. Immunogenicity was assessed through Days 29 (primary) and 181 (exploratory) by nAb responses against vaccine-matched XBB.1.5 variant and emerging variants of JN.1 lineage. Results for the monovalent XBB.1.5 vaccine are described.

**Results:**

Fifty adults ≥18 years were randomized to receive mRNA-1273.815 (median [range] age, 55 [21-84] years; 68.0% with evidence of prior SARS-CoV-2 infection). Through Day 181, no vaccine-related deaths, SAEs, MAAEs, AEs leading to withdrawal, or AESIs were reported. At Day 29, mRNA-1273.815 substantially increased nAb titers against XBB.1.5 by 17.5-fold from pre-booster levels, remaining 5.2-fold above pre-booster levels at 6 months after vaccination (Figure). Vaccination also induced nAb responses against divergent JN.1, JN.1.23, and KP.2 variants at Days 29 and 181; however, the GMT and fold-rise for these variants was lower at all timepoints than for those against XBB.1.5 (Figure). Similar trends were seen regardless of prior SARS-CoV-2 status or intercurrent SARS-CoV-2 infection.

**Conclusion:**

mRNA-1273.815 had a safety profile consistent with previously authorized mRNA-1273 vaccines and induced durable nAb responses against XBB.1.5. through 6 months. Responses were lower against the divergent JN.1 lineage variants.

**Disclosures:**

**Frances Priddy, MD, MPH**, Moderna, Inc.: Employee|Moderna, Inc.: Stocks/Bonds (Private Company) **Nichole McGhee, B.S.**, Moderna, Inc.: Employee|Moderna, Inc.: Stocks/Bonds (Private Company) **Spyros Chalkias, MD**, Moderna, Inc.: Employee|Moderna, Inc.: Stocks/Bonds (Private Company) **Bethany Girard, PhD**, Moderna, Inc.: Employee|Moderna, Inc.: Stocks/Bonds (Private Company) **Darby Wang, BS**, Moderna, Inc.: Employee|Moderna, Inc.: Stocks/Bonds (Private Company) **Elizabeth de Windt, MPH**, Moderna, Inc.: Employee|Moderna, Inc.: Stocks/Bonds (Private Company) **Darin Edwards, Ph.D.**, Moderna, Inc.: Employee|Moderna, Inc.: Stocks/Bonds (Private Company) **Jing Feng, M.S.**, Moderna, Inc.: Employee|Moderna, Inc.: Stocks/Bonds (Private Company) **David C. Montefiori, PhD**, Moderna, Inc.: Grant/Research Support **Jacqueline Miller, MD**, Moderna, Inc.: Employee|Moderna, Inc.: Stocks/Bonds (Public Company) **Rituparna Das, M.D.**, Moderna, Inc.: Employee|Moderna, Inc.: Stocks/Bonds (Public Company)

